# Research on the Preparation and Properties of High Belite Sulphoaluminate Cement (HBSAC) Based on Various Industrial Solid Wastes

**DOI:** 10.3390/ma12091510

**Published:** 2019-05-09

**Authors:** Dunlei Su, Gongbing Yue, Qiuyi Li, Yuanxin Guo, Song Gao, Liang Wang

**Affiliations:** 1 School of Civil Engineering, Qingdao University of Technology, Qingdao 266033, China; sudunlei@163.com (D.S.); gaosong727@126.com (S.G.); 2 School of Architectural Engineering, Qingdao Agricultural University, Qingdao 266109, China; guoyuanxin2019@163.com (Y.G.); jiangongwl_2019@163.com (L.W.)

**Keywords:** industrial solid wastes, petroleum coke desulfurization slag, high belite sulphoaluminate cement, sintering temperature, property

## Abstract

In this study, a variety of industrial solid wastes, including petroleum coke desulfurization slag, fly ash and carbide slag with natural resource bauxite, were used as raw materials to prepare high belite suphoaluminate cement, which contains a certain CaSO_4_ content without adding natural gypsum to the clinker. The sintering temperature, mineral composition, and the physical and mechanical properties of the cement clinkers were investigated. The techniques adopted included a comprehensive thermal analysis (DSC-TG), X-ray diffraction (XRD), X-ray fluorescence (XRF) and scanning electron microscopy (SEM). The results revealed that it is completely feasible to prepare high belite sulphoaluminate cement with the various industrial solid wastes mentioned above and the utilization rate of the solid wastes is up to 80%. The sintering temperature ranges from 1225 °C to 1350 °C, and the optimal sintering temperature is approximately 1300 °C. The clinkers prepared at 1300 °C set and harden quickly and have a slightly higher water requirement of normal consistency. The mechanical strength is greatly affected by the CaSO_4_ and 3CaO·3Al_2_O_3_·CaSO_4_ contents and the most reasonable CaSO_4_ content is 15%.

## 1. Introduction

Sulphoaluminate cement (SAC) is the third series of cements after Portland cement and aluminate cement, and it has the characteristics of early strength, high strength, high impermeability, high frost resistance, corrosion resistance, low alkali and low production energy consumption; SAC is widely used in rapid construction, rapid repair, winter construction, marine environments and underground engineering [1,2,3]. Its clinker is mainly composed of C_4_A_3_S (ye’elimite, 3CaO·3Al_2_O_3_·CaSO_4_) and β-C_2_S (belite, 2CaO·SiO_2_). Among them, C_4_A_3_S is an early strength mineral that can quickly hydrate to AFt (ettringite, 3CaO·Al_2_O_3_·3CaSO_4_·32H_2_O) in the presence of gypsum to provide early strength [4]. Compared with C_3_S (alite, 3CaO·SiO_2_), the main mineral component of Portland cement, the formation temperature of C_4_A_3_S and the content of CaO are lower [5]. It not only saves a great deal of coal, power and limestone resources but also reduces CO_2_ emissions [6]. However, SAC has many defects, such as the low content and slow hydration of C_2_S, resulting in no significant increase in the later strength of SAC [7,8]; the formation of C_4_A_3_S requires a large amount of natural gypsum and high-quality aluminum resources. 

As a new type of SAC, high belite sulphoaluminate cement (HBSAC) has an optimized mineral composition achieved by reducing the C_4_A_3_S content and increasing the C_2_S content [1,9], which effectively guarantees the development of late strength of the cement, reduces the amount of natural gypsum and alumina resources, and provides the conditions for the utilization of low-quality aluminum resources. Generally, limestone, bauxite or ball clay and gypsum are still used as raw materials for the preparation of HBSAC. However, after long-term and large-scale exploitation, the aforementioned raw materials are increasingly scarce, and the use of various industrial solid wastes as alternative raw materials has become the focus of researchers. El-Alfi et al. [10] succeeded in preparing HBSAC by sintering marble sludge waste, kaolinite and gypsum at 1200–1250 °C for 1 h without using limestone. Xue et al. [11] and Adolfsson et al. [12] used different types of steelmaking slag to get HBSAC, respectively. Sahu et al. [13] prepared HBSAC by calcining fly ash, limestone and gypsum at 1200 °C for 30 min without using any natural aluminum resources. Xu et al. [14] successfully obtained HBSAC from coal gangue, which not only made full use of the silicon and aluminium components in coal gangue but also utilized the low calorific value fuel function of coal gangue. Zhang et al. [15] applied titanium tailings to the preparation of HBSAC, showing that the high content of TiO_2_ has no adverse effect on cement formation, and proper amount of TiO_2_ can promote the formation of C_2_S. Huang et al. [16] used phosphogypsum to produce HBSAC as a raw material for the HBSAC clinker and added gypsum, which reduced the consumption of natural gypsum. Li et al. [17] employed lithium mica slag, whose main components are CS and Ca(AlO_2_)_2_, and low grad bauxite to prepare HBSAC with limestone and bauxite at 1270–1320 °C. Wang et al. [18] substituted natural gypsum for desulfurized gypsum, bauxite for red mud partially or fully to prepare HBSAC with limestone and bauxite at 1300 °C, the desulfurization gypsum and red mud can make up 70%–90% by mass of the total raw materials; and red mud as a resource of iron and aluminium in the preparation of HBSAC can also be seen in reference [19]. Tangshan Polar Bear Building Materials Co., Ltd. [20] adopted fly ash and desulfurization gypsum to produce fast-setting and quick-hardening HBSAC with other natural resources, which is popular in the Chinese cement market and has been applied in the Qingdao Airport and oilfields in Dongying, achieving good economic benefits. In addition, Li et al. [21] obtained HBSAC by microwave sintering with fly ash, baghouse dust and scrubber sludge as raw materials at 1150 °C for 10 min, showing obvious advantages of energy saving; Rungchet et al. [22] prepared HBSAC with fly ash, Al-rich sludge and flue gas desulfurization gypsum via the hydrothermal-calcination method at a calcination temperature of only 1050 °C, and the resulting products were friable and easy to grind, leading to energy saving. Overall, these works showed the feasibility to prepare HBSAC using various industrial wastes as raw materials. However, it needs to be emphasized that there is a large class of industrial solid waste—petroleum coke desulfurization slag—which is rarely considered but needs to be developed urgently.

In the coastal areas of Shandong Province, China, the crude oil for large refineries is mainly imported from the Middle East. The sulphur, nitrogen and metal element content in heavy and inferior crude oil are relatively high. Petroleum coke can only be desulfurized as fuel by circulating it in fluidized bed boilers. The desulfurization gypsum formed after this treatment is mixed with other combustion products to form petroleum coke desulfurization slag, whose main components are CaO and CS (anhydrite, CaSO_4_) [23]. Taking the Qingdao Refinery as an example, the discharge of petroleum coke desulfurization slag is more than 500,000 tons per year. However, its direct applications as building materials are limited because of the poor cement stability caused by CaO and CS in ordinary Portland cement and the secondary pollution caused by the release of SO_2_. At present, most of the slag is simply landfilled or stored in the open, which not only wastes land and resources but also pollutes the environment. Considering the preparation of HBSAC, it seems completely feasible to use petroleum coke desulfurization slag instead of limestone, which produces a large amount of CO_2_, and gypsum, which is a scarce resource, to provide calcium and sulfur elements needed for the clinker mineral formation. In addition, by controlling the amount of petroleum coke desulfurization slag, the residual CS in the clinker can replace the gypsum in the cement clinker admixture, which can further save natural gypsum.

Therefore, in this study, petroleum coke desulfurization slag was selected as the raw material for calcium and sulphur. To improve the comprehensive utilization of solid wastes, fly ash was selected as the raw material for aluminum and silica, while carbide slag (the waste residue from the hydrolysis of calcium carbide to acetylene) and bauxite were used as supplementary materials for calcium, aluminum and silica. Based on the materials mentioned above, HBSAC clinkers of varying CS contents were prepared, and their sintering temperature, mineral composition, physical and mechanical properties were also systematically studied.

## 2. Experimental Details

### 2.1. Raw Materials

The raw materials used in this study were petroleum coke desulfurization slag, fly ash, carbide slag and bauxite. The petroleum coke desulfurization slag was collected from the Sinopec Qingdao Refinery, Qingdao, China, the fly ash (Class I) from the Qingdao Municipal Concrete Industry Co., Ltd., Qingdao, China, the carbide slag from the Qingdao Qingxin Building Materials Co., Ltd., Qingdao, China, and bauxite from the Gongyi Wanying Environmental Protection Materials Co., Ltd., Gongyi, China. The main chemical composition of each raw material was measured by an X-ray fluorescence spectrometer (1800 type, Shimadzu Co., Kyoto, Japan) and are shown in Table 1. 

### 2.2. Mix Design

In the process of developing a mixing design, it is assumed that the mineral formation reactions proceed as follows: (1)4CaO + Al_2_O_3_ + Fe_2_O_3_→C_4_AF (tetracalcium aluminoferrite, 4CaO·Al_2_O_3_·Fe_2_O_3_); (2)3CaO + 3Al_2_O_3_ + CS→C_4_A_3_S; (3)2CaO + SiO_2_→β-C_2_S. Then, the mineral content of the target clinker should be set. In this study, a total of nine ratios in three series were designed, as shown in Table 2. Based on the mineral composition of the clinker, the chemical composition of the clinker can be calculated, and then the amounts of raw materials can be deduced. Meanwhile, it is necessary to control the alkalinity coefficient of the clinkers (i.e., *C_m_*) [1] so that it is not less than 1.0, as it will also affect the raw material amounts, especially for the calcareous materials.

### 2.3. Preparation of Clinkers

**Figure 1:**
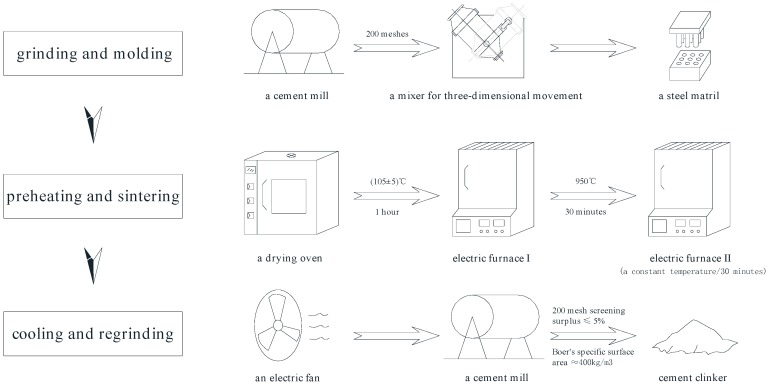
Processing diagram of cement clinker preparation.

The preparation of clinkers by using various industrial solid wastes can be divided into three steps: grinding and molding, preheating and sintering, and cooling and regrinding. The specific operations are as follows: (1) All of the raw materials were ground by a cement mill to pass through a 200 mesh square hole sieve and then mixed evenly and pressed into a steel matrix to form cylindrical test samples with a diameter of 15 mm and a height of 17 mm. (2) Samples were dried for 1 h in a drying oven at a constant temperature of 105 ± 5 °C and then preheated for 30 min in a high temperature electric furnace (SX-8-16 type, Beijing Ever Bright Medical Treatment Instruments Co., Ltd., Beijing, China) at a constant temperature of 950 °C and then sintered for 30 min in another high temperature electric furnace at a constant temperature. (3) The samples were removed and air cooled [24] and then ground to pass through a 200-mesh square hole sieve with a surplus of less than 5% or a Boer’s specific surface area of approximately 400 kg/m^3^. The processing diagram is shown in Figure 1.

### 2.4. Test Methods

A comprehensive thermal analyzer (SDT Q600 type, TA Instruments Co., New Castle, DE, U.S.A.) was employed to measure the weight change, the heat absorption and exothermy of raw materials during the sintering process, while the weight was monitored from room temperature (approximately 20 °C) to 1400 °C at a heating rate of 20 °C/min while continuously purging with N_2_. The mineral phase and hydration products of the clinkers were detected by an X-ray diffraction instrument (D8 advance type, Bruker Co., Karlsruhe, Germany) with the working conditions as follows: Cu target, voltage at 40 kV, current at 40 mA, 2-Theta scanning ranges from 5–60°, step width of 0.02° and a residence time of 0.05 s. The main chemical composition of the clinkers were measured by an X-ray fluorescence spectrometry (1800 type, Shimadzu Co., Kyoto, Japan). The basic physical properties of the clinkers, such as the water consumption at standard consistency and setting time, were determined according to the “Test methods for water requirement of normal consistency, setting time and soundness of the portland cement” (GB/T 1346-2011, China). According to the “Method of testing cements-Determination of strength” (GB/T 17671-1999, China) and the “Sulphoaluminate cement” (GB 20472-2006, China), the mechanical strength was tested, and the cement mortar pieces used were 40 × 40 × 160 mm samples prepared under a water–cement ratio of 0.52, which were cured for 1, 3, 7 and 28 days, respectively. A scanning electron microscope (JSM-7500F type, JEOL Co., LTD., Tokyo, Japan) with a working voltage of 5.0 kV was used to analyze the micromorphology of the clinker minerals and hydration products. The clinker samples were powdered, and the samples of the hydration products used were small pieces, which were sprayed with gold before testing.

## 3. Results and Discussion

### 3.1. Sintering Temperature of Clinkers

**Figure 2:**
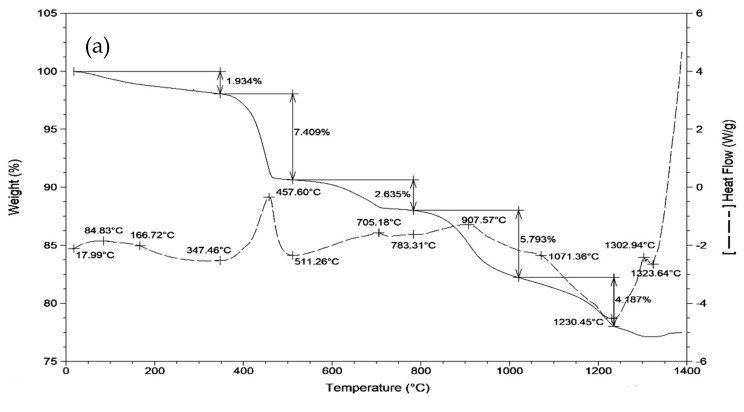

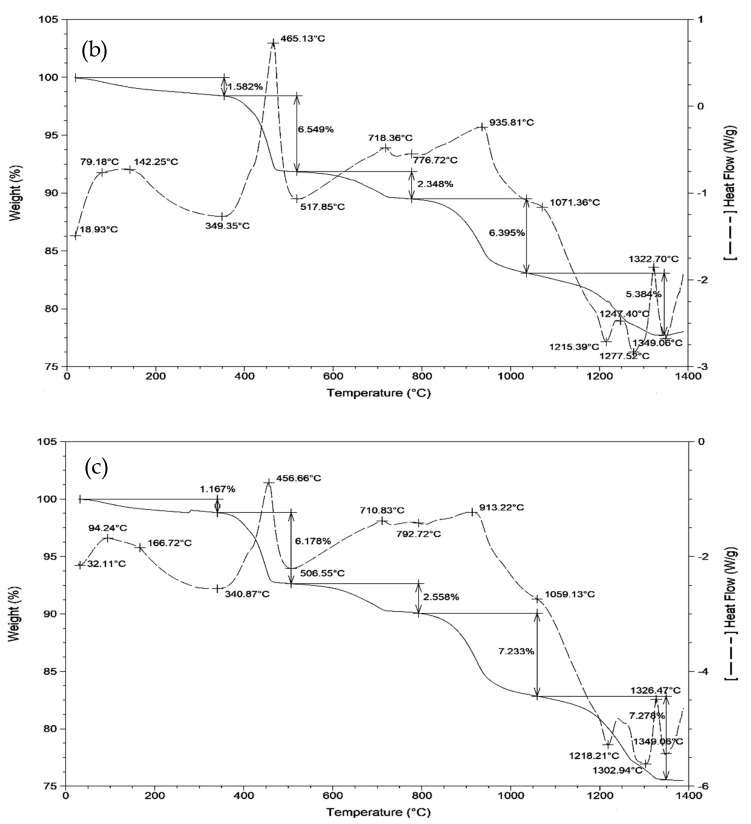
Comprehensive thermal analysis (DSC-TG) curves of the mixtures: (**a**) I-1; (**b**) II-1; (**c**) III-1.

**Figure 3:**
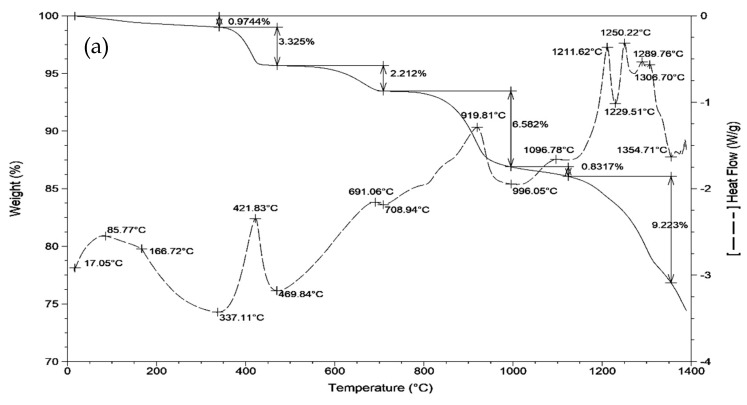

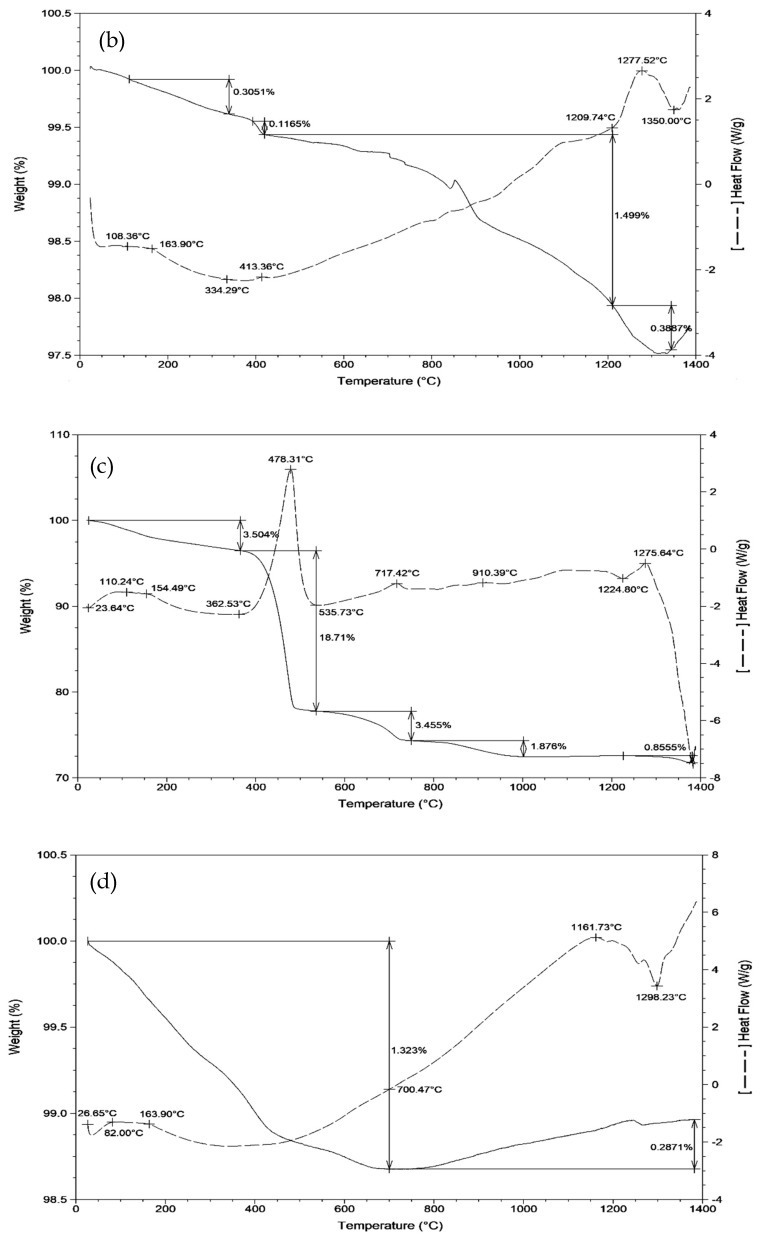
Comprehensive thermal analysis (DSC-TG) curves of the raw materials: (**a**) petroleum coke desulfurization slag; (**b**) fly ash; (**c**) carbide slag; (**d**) bauxite.

Comprehensive thermal analysis can characterize the mass and heat changes in the clinker formation process to approximate preliminarily the temperature of the reaction among the raw materials. As shown in Figure 2, the DSC-TG curves of the mixtures (i.e., samples I-1, II-1 and III-1) show a similar change rule. Combined with the DSC-TG curves of the raw materials in Figure 3, it is not difficult to obtain some meaningful results: when the temperature is below 200 °C, the endothermic peaks and mass loss in the DSC-TG curves should originate from the evaporation of the physical water in all the raw materials; when temperature reaches 400–500 °C, an endothermic peak should be caused by the decomposition of the Ca(OH)_2_ in the petroleum coke desulfurization slag (formed by the CaO absorbing water from the air) and in the carbide slag; at approximately 710 °C, 910 °C and 1050 °C, there are different sized endothermic peaks with mass loss, which are still caused by the petroleum coke desulfurization slag and carbide slag. It appears that there was no new mineral formation before 1050 °C, and in this process, the mass loss and heat change of the fly ash and bauxite were also not significant. The reason may be that these two raw materials have excellent resistance to elevated temperatures. After 1050 °C, all the raw materials were involved in the reaction, including a series of chemical changes such as the decomposition of the CS, the formation and decomposition of the C_2_AS (gehlenite, 2CaO·Al_2_O_3_·SiO_2_) [21], C_4_A_3_S, C_5_S_2_S (ternesite, 4CaO·2SiO_2_·CaSO_4_) [21,25,26] and β-C_2_S. The comprehensive effect was to form exothermic peaks in the 1050–1350 °C range, which was also used preliminarily as the sintering temperature.

**Figure 4:**
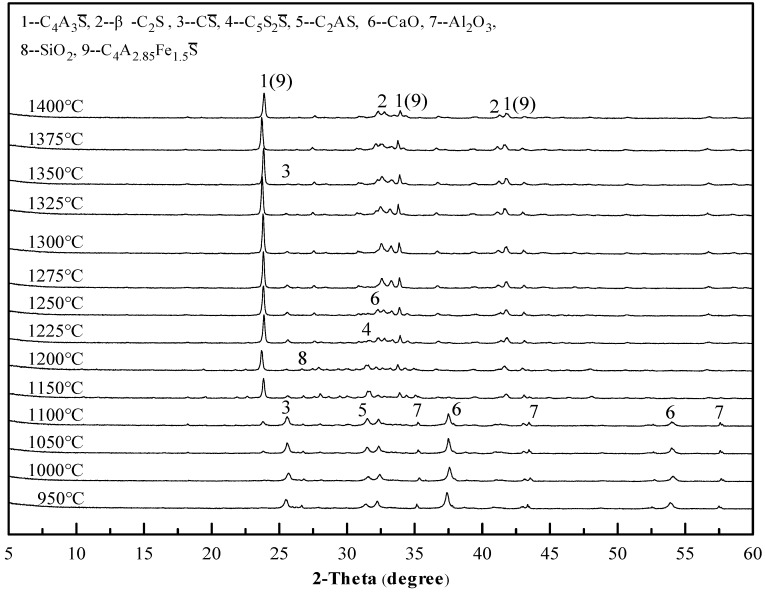
X-ray diffraction (XRD) patterns of the sample II-1 cement clinker at different temperatures.

To determine the clinker sintering temperature and assess the formation of products more accurately, an X-ray diffraction analysis was carried out. The X-ray diffraction (XRD) patterns of the sample II-1 cement clinker at different temperatures are given in Figure 4. When the sintering temperature is in the range of 950–1100 °C, the main products are CS, C_2_AS, CaO, Al_2_O_3_ and SiO_2_, and C_4_A_3_S begins to form in a small amount at 1050 °C, which is consistent with the results of the comprehensive thermal analysis. The temperature rises to 1150–1225 °C, C_4_A_3_S gradually forms in large quantities; additionally, the C_2_AS disappears and C_5_S_2_S appears, and the sintering products are mainly C_4_A_3_S and C_5_S_2_S. As the temperature continues to rise to 1250–1300 °C, the C_5_S_2_S decomposes, and β-C_2_S gradually forms; the main products are C_4_A_3_S, β-C_2_S and CS. At the same time, the intensity of the C_4_A_3_S and β-C_2_S diffraction peaks increases with the increase in temperature, while that of the CS decreases, indicating that the increase in temperature promotes high temperature reactions. As the temperature increases again, the intensity of the diffraction peaks of each product begins to decrease, and the CS diffraction peaks are hardly observed, especially after 1350 °C. This finding indicates that the increase in temperature has caused the decomposition of the mineral phases. It can be seen that the sintering temperature of the sample II-1 cement clinker ranges from 1250 °C to 1350 °C, and the optimum sintering temperature is 1300 °C. It is worth pointing out that the iron element did not form the anticipated C_4_AF but mainly formed C_4_A_2.85_Fe_1.5_S(3CaO·2.85Al_2_O_3_·1.5Fe_2_O_3_·CaSO_4_) by solid solution in the C_4_A_3_S [25], regardless of the sintering temperature. 

**Figure 5:**
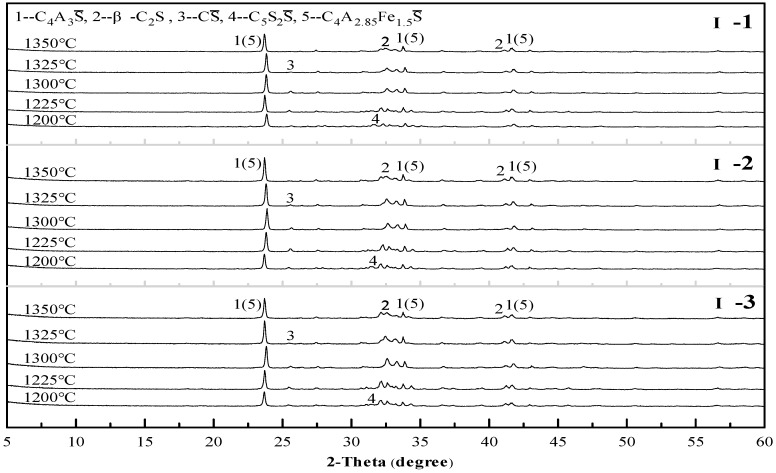
X-ray diffraction (XRD) patterns of the Series I cement clinkers at different temperatures.

**Figure 6:**
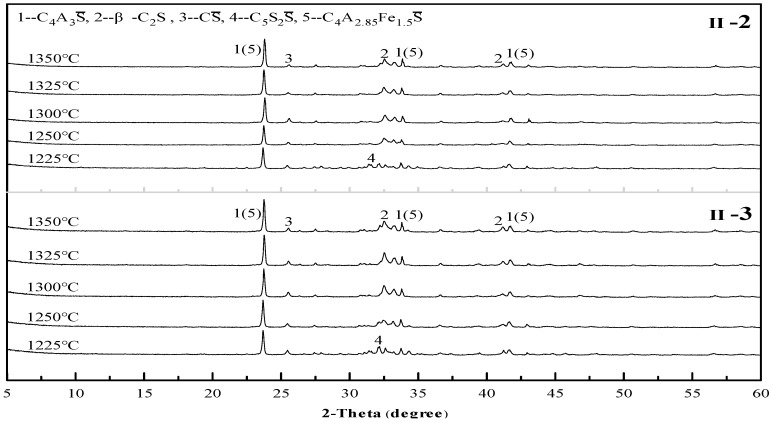
X-ray diffraction (XRD) patterns of the Series II cement clinkers at different temperatures.

**Figure 7:**
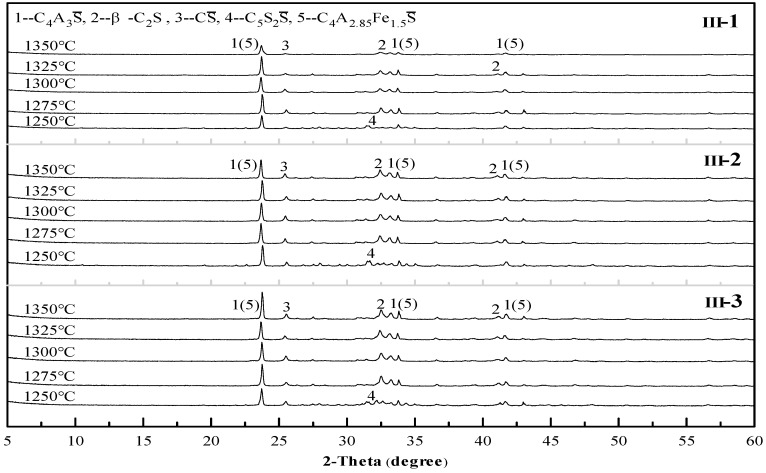
X-ray diffraction (XRD) patterns of the Series III cement clinkers at different temperatures.

In accordance with the same principle of analysis, all the other mix proportions besides sample II-1 were tested to study the influence of the mix proportion on the sintering temperature. Some of the results are shown in Figure 5, Figure 6 and Figure 7. As revealed in these figures, the sintering temperature ranges of the Series I, II, and III cement clinkers are approximately 1225–1325 °C, 1250–1350 °C, and  1275–1350 °C, respectively. Thus, with the increase of the CS content in the mix design, the lower limit of the sintering temperature increases gradually, which is controlled by the decomposition of C_5_S_2_S, and the range of the sintering temperature is basically maintained at 100 °C. In addition, the optimal sintering temperature of all the mix proportions is approximately 1300 °C.

### 3.2. Mineral Composition of Clinkers

**Figure 8:**
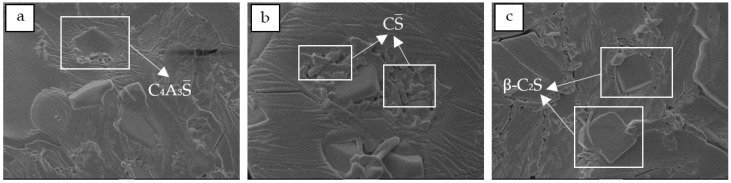
Micromorphology of the sample II-1 cement clinker: (**a**) C_4_A_3_S; (**b**) CS; (**c**) β-C_2_S.

The micromorphology of the minerals in the clinker was observed by scanning electron microscopy (SEM), and the mineral composition was qualitatively analyzed. Taking the sample II-1 cement clinker prepared at 1300 °C as an example, the results are shown in Figure 8. The cement clinker system was mainly composed of tabular C_4_A_3_S, blocky granular β-C_2_S, and radical and needle-bar CS. The mineral composition is basically consistent with the X-ray diffraction analysis. 

In addition to the qualitative analysis of the mineral composition, a quantitative analysis of the clinker minerals was also carried out. The chemical composition of the cement clinkers prepared at 1300 °C was measured as shown in Table 3. The clinker mineral content is calculated according to Bogue’s equation [27]. However, because of the existence of residual CS and C_4_A_2.85_Fe_1.5_S and the absence of C_4_AF in the cement clinker, Bogue’s equation needs to be modified [28,29,30,31], as shown in Equations (1)–(4), while the alkalinity coefficient is also been modified, as shown in Equation (5).

ω(C_2_S) = 2.87ω(SiO_2_) (1)

ω(C_4_A_2.85_Fe_1.5_S) = 3.48ω(Fe_2_O_3_) (2)

ω(C_4_A_3_S) = 1.99[ω(Al_2_O_3_) − 1.21ω(Fe_2_O_3_)] (3)

ω(CS) = 1.70ω(SO_3_) − 0.44ω(Al_2_O_3_) − 0.02ω(Fe_2_O_3_) (4)

*C_m_* = [ω(CaO) − 0.7ω(TiO_2_) − 0.41ω(CS)]/{0.73[ω(Al_2_O_3_) − 1.21ω(Fe_2_O_3_)] + 0.93ω(Fe_2_O_3_) + 1.87ω(SiO_2_)} (5)

As revealed in Table 4, the actual mineral content of the cement clinker is not significantly different from the design content in Table 2. More specifically, the content error of the C_4_A_3_S and β-C_2_S is approximately 5%, while that of the CS is within 2%, even the content error between the C_4_A_2.85_Fe_1.5_S and C_4_AF is less than 5%. In addition, the alkalinity coefficients of all the clinkers are slightly greater than 1.0, indicating that the CaO in the raw materials can meet the requirements for the formation of various useful minerals. 

By the qualitative and quantitative analyses mentioned above, the mineral composition of the clinkers basically conforms to the expected product, and it is indirectly proven that the mix design and sintering conditions are reasonable.

### 3.3. Physical and Mechanical Properties of the Clinkers

The physical and mechanical properties are the most basic elements of cement performance. In this study, the water requirement of normal consistency, setting time and mechanical strength of the clinkers with different proportions prepared at 1300 °C were tested. As shown in Table 5, the water requirement of normal consistency, which was prepared by using various solid wastes, is in the range of 36%–40%, which is slightly larger than that of typical clinkers (approximately 30%). The initial setting time and the final setting time are 17–25 min and 23–40 min, respectively. The setting time is relatively short, which is more suitable for projects involving emergency rescue and repair. Further analysis shows that the water requirement of normal consistency decreases and the setting time prolongs as the C_4_A_3_S content decreases in each series. In view of the fact that the early hydration of HBSAC is dominated by C_4_A_3_S [32,33,34], it can be considered that the C_4_A_3_S content plays a direct and decisive role in the water requirement of normal consistency and in the setting time. Comparing sample I-1 with II-1, or sample I-2, II-2 with III-1, or sample I-3 with III-2, it is easy to find that the water requirement of normal consistency decreases and the setting time shortens as the CS content increases under the same C_4_A_3_S content, which indicates that an increase in the CS content in the range of 10%–20% helps to accelerate the C_4_A_3_S hydration.

**Figure 9:**
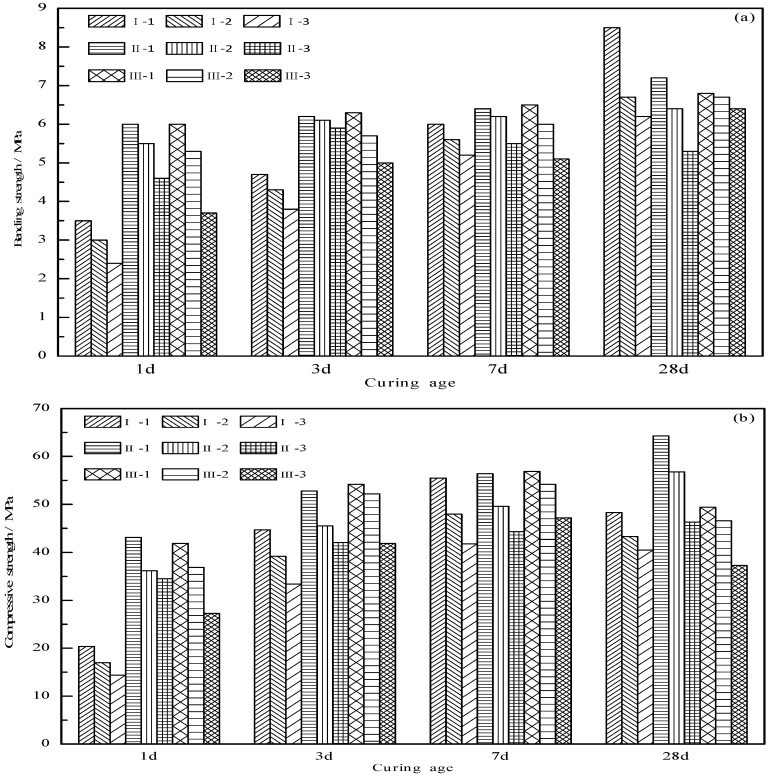
Mechanical strength of the cement clinkers: (**a**) bending strength; (**b**) compressive strength.

Figure 9 shows the mechanical strength of the cement clinkers measured by using the mortar pieces at a water-cement ratio of 0.52. As shown in Figure 9a, the bending strength of each series increases as the curing age increases, and the bending strength increases rapidly within 3 days and slows down after 3 days. For example, as the lowest early bending strengths of all the mixtures, the bending strengths of sample I-3 at 1, 3, 7 and 28 days are 2.4 MPa, 3.8 MPa, 5.2 MPa and 6.2 MPa, respectively, while the strength increases at 3, 7 and 28 days are 58%, 37% and 20%, respectively. Sample II-2, as the middle early bending strength sample of all the mixtures, has bending strengths of 5.5 MPa, 6.1 MPa, 6.2 MPa and 6.4 MPa, respectively at 1, 3, 7 and 28 days, and strength increases of 11%, 2% and 3%, respectively at 3, 7 and 28 days. Of all the mixtures, sample III-1 has the highest early bending strength; its bending strengths at 1, 3, 7 and 28 days are 6.0 MPa, 6.3 MPa, 6.6 MPa and 6.8 MPa, respectively, while the strength increases at 3, 7 and 28 days are 5%, 5%, and 3%, respectively. In addition, there are several other findings: (1) The increase in the bending strength of the samples in Series II and III at 7 days has decreased to less than 5%, while that of the samples in Series I is approximately 30%, which indicates that the increased CS content in the clinker has an obvious positive effect on the development of early bending strength [35]. (2) Comparing the bending strength of samples I-1 and II-1 (or samples I-2, II-2 and III-1) at different curing ages, it can be seen that under the same C_4_A_3_S content, the bending strength at 1, 3, and 7 days increases as the CS content increases. However, there is no similar trend for the bending strength at 28 days, which shows from another perspective that the increased CS content in the clinker has an obvious positive effect on the development of early bending strength. The bending strength at 28 days decreases with the β-C_2_S content decreases, which means that the hydration of β-C_2_S is the main factor for the strength development of the cement clinker in the later stage [36]. (3) During the entire 28-day curing process, the bending strength of each series decreases as the C_4_A_3_S content decreases. The β-C_2_S content does not change the development trend of the later strength, indicating that the hydration of β-C_2_S in the clinker is not so strong that the bending strength of the cement clinkers at 28 days is still dominated by the C_4_A_3_S content.

**Figure 10:**
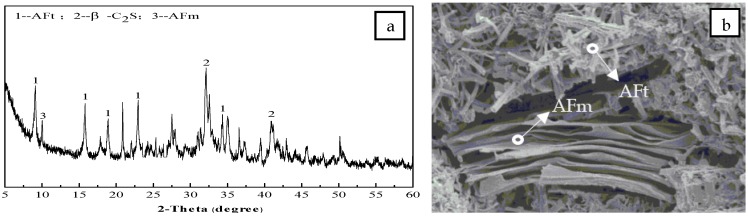
X-ray diffraction (XRD) patterns and scanning electron microscopy (SEM) micromorphology of the hydration products of the Series I cement clinker at 28 days: (**a**) XRD patterns; (**b**) SEM micromorphology.

In terms of compressive strength, as shown in Figure 9b, there are obvious differences among the three series with the increase in the curing age. The compressive strength of Series I and III samples decreases at 28 days, while that of Series II samples maintains good growth during the whole curing age. It can be seen that the CS content in the clinker is not as high as possible, a lower or higher CS content in the clinker may have a negative impact on the compressive strength. The reasons are as follows: when the CS content is lower, the AFt phase formed in the early hydration stage is partly converted to the AFm (ettringite, C_3_A·CaSO_4_·12H_2_O) phase in the later hydration stage [37,38,39], as shown in Figure 10, which results in a decrease in the compressive strength; when the CS content is higher, the rate of the AFt phase formation in the early hydration stage is too fast, and the crystal structure continues to grow in the later hydration stage, which causes expansion and microcrack damage [26], and also results in a decrease in the compressive strength. According to the results of this experiment, 15% is the optimal CS content in this kind of cement clinker; under this content, the lowest compressive strengths are 42.1 MPa, 46.3 MPa, and the highest are 52.8 MPa, 64.3 MPa at 3 days and 28 days, respectively. The change rule of the compressive strength before 28 days is the same as that of the bending strength: the strength increases rapidly in 3 days but slows down after 3 days, and the increase of the CS content in the clinker has an obvious positive effect on the development of the early compressive strength. The specific strength value can be referred to in Figure 9b, and will not be repeated here. 

**Figure 11:**
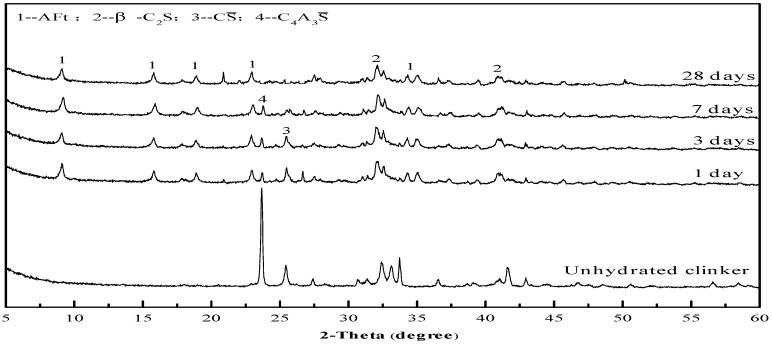
X-ray diffraction (XRD) patterns of the hydration products of the Series II cement clinker at different curing ages.

**Figure 12:**
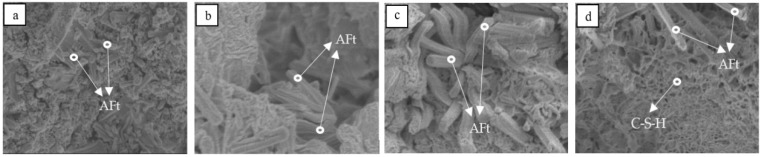
SEM micromorphology of the hydration products of the Series II cement clinker at different curing ages: (**a**) 1 day; (**b**) 3 days; (**c**) 7 days; (**d**) 28 days.

The formation of the hydration products can reasonably reflect the development of the cement strength. As shown in Figure 11, the main hydration product of the cement is the AFt phase, and the C_4_A_3_S hydration is the main hydration reaction during the 7 days, while CS is gradually consumed. The C_2_S hydration is slow, and C_2_S diffraction peaks in the XRD patterns still change slightly at 28 days. In Figure 12, the needle bar phase is the AFt phase, and at the initial hydration stage, the AFt phase are fine needle rods, which gradually develop into thick needle rods as the curing age increases. At the later hydration stage, the flocculent C-S-H gel phase appears and intercalates with the needle AFt phase, which makes the structure of the cement paste denser and further increases the strength. Thus, the formation process of the hydration products coincides with the strength development law as the curing age increases. In addition, the XRD patterns and SEM micromorphology of the hydration products of the Series I cement clinker before 28 days and the Series II cement clinker during the whole curing age, are basically similar to those of the Series II cement clinker and are not listed here.

## 5. Conclusions

It is completely feasible to prepare HBSAC with petroleum coke desulfurization slag, fly ash, carbide slag and bauxite synergistically. The cement clinkers prepared contain 10%–20% of CS without adding additional natural gypsum. The comprehensive utilization rate of the solid waste is up to 80%. 

The sintering temperature range of the cement clinkers prepared with solid wastes such as petroleum coke desulfurization slag and fly ash is 1225–1350 °C, and with a 5% gradient increase in the cement clinker CS content, the lower limit of the sintering temperature increases by a 25 °C gradient, which is caused by the increase in the C_5_S_2_S decomposition temperature. The optimal sintering temperature is approximately 1300 °C. 

The mineral composition of the cement clinkers prepared with solid wastes such as petroleum coke desulfurization slag and fly ash is mainly C_4_A_3_S, β-C_2_S and CS. The iron element does not form the anticipated C_4_AF but mainly forms C_4_A_2.85_Fe_1.5_S by solid solution in C_4_A_3_S. The difference between the actual mineral content and the designed mineral content of the clinkers is within a reasonable range.

The cement clinkers prepared at 1300 °C set and harden quickly and have a slightly higher water requirement of normal consistency. The water requirement of normal consistency is 36%–40%, and the initial setting time and final setting time are 17–23 min, 25–40 min, respectively. The mechanical strength is greatly affected by the clinker CS and C_4_A_3_Scontents, and the optimal CS content is 15%. In terms of the hydration products of the cement clinkers, the AFt and C-S-H gel are still the main phases, which are formed by C_4_A_3_S hydration at the early stage and C_2_S hydration at the later stage, respectively.

## Figures and Tables

**Table 1:** Chemical composition of the raw materials, wt.%.

Raw Material	CaO	Al_2_O_3_	SiO_2_	Fe_2_O_3_	SO_3_	MgO	TiO_2_	LOI	∑
**Petroleum coke desulfurization slag**	52.93	0.96	4.56	1.13	30.12	2.03	0.00	7.48	99.21
**Fly ash**	7.86	27.45	52.56	4.24	1.26	1.12	0.99	1.73	97.21
**Carbide slag**	66.02	1.47	4.61	0.68	1.97	0.25	0.00	24.62	99.62
**Bauxite**	0.51	64.07	14.53	0.88	0.00	15.38	2.56	1.03	98.96

**Table 2:** Design of the mineral composition of the clinker and the proportion of the raw materials, wt.%.

Series	Group	Clinker Minerals				Raw Materials			
		C_4_AF	C_4_A_3_S	C_2_S	CS	Petroleum Coke Desulfurization Slag	Fly Ash	Carbide Slag	Bauxite
**I**	**1**	5	40	45	10	31.8	12.0	35.1	21.1
	**2**	5	35	50	10	29.0	16.0	38.6	16.4
	**3**	5	30	55	10	27.9	19.3	40.7	12.1
**II**	**1**	5	40	40	15	39.2	10.8	29.2	20.8
	**2**	5	35	45	15	37.5	14.3	32.0	16.2
	**3**	5	30	50	15	35.2	18.4	35.2	11.2
**III**	**1**	5	35	40	20	47.2	9.9	23.2	19.7
	**2**	5	30	45	20	45.0	13.8	26.4	14.8
	**3**	5	25	50	20	42.5	17.9	29.8	9.8

**Table 3:** Chemical composition of the cement clinkers, wt.%.

Series	Group	CaO	Al_2_O_3_	Fe_2_O_3_	SiO_2_	SO_3_	MgO	TiO_2_	∑
**I**	**1**	45.76	20.36	2.36	13.95	10.55	3.20	0.73	96.91
	**2**	47.23	18.09	2.44	15.46	9.86	2.42	0.64	96.14
	**3**	48.65	15.97	2.57	17.08	9.29	1.71	0.55	95.82
**II**	**1**	45.34	20.78	2.32	12.56	13.67	3.04	0.68	98.39
	**2**	46.75	18.24	2.47	14.13	12.93	2.30	0.60	97.42
	**3**	48.04	16.02	2.63	16.07	12.27	1.47	0.50	97.00
**III**	**1**	45.83	18.25	2.15	12.63	15.88	3.00	0.77	98.51
	**2**	47.26	16.13	2.29	14.23	15.39	2.20	0.68	98.18
	**3**	48.75	13.88	2.45	15.64	14.65	1.37	0.58	97.32

**Table 4:** Mineral content, wt.% and alkalinity coefficient of the cement clinkers.

Series	Group	C_4_A_3_S	β-C_2_S	C_4_A_2.85_Fe_1.5_S	CS	∑	*C_m_*
**I**	**1**	34.83	40.04	8.21	8.93	92.01	1.01
	**2**	30.12	44.37	8.49	8.75	91.73	1.02
	**3**	25.59	49.02	8.94	8.71	92.26	1.02
**II**	**1**	35.77	36.05	8.07	14.05	93.94	1.01
	**2**	30.35	40.55	8.60	13.91	93.41	1.02
	**3**	25.55	46.12	9.15	13.76	94.58	1.00
**III**	**1**	31.14	36.25	7.48	18.92	93.79	1.01
	**2**	26.58	40.84	7.97	19.02	94.41	1.01
	**3**	21.72	44.89	8.53	18.75	93.89	1.03

**Table 5:** Physical properties of cement clinkers.

Series	Group	Water Requirement of Normal Consistency (wt.%)	Initial Setting Time (min)	Final Setting Time (min)
**I**	**1**	39.5	21.80	35.95
	**2**	39.0	23.17	37.75
	**3**	38.5	24.50	39.53
**II**	**1**	37.5	20.10	30.60
	**2**	37.0	21.08	31.58
	**3**	36.5	22.75	32.08
**III**	**1**	37.0	17.05	23.45
	**2**	36.5	18.18	26.42
	**3**	36.0	19.38	28.20

## Abbreviations

The following abbreviations are used in this manuscript:

SAC	sulphoaluminate cement
HBSAC	high belite sulphoaluminate cement
C_4_A_3_S	3CaO·3Al_2_O_3_·CaSO_4_
β-C_2_S	2CaO·SiO_2_
CS	CaSO_4_
C_2_AS	2CaO·Al_2_O_3_·SiO_2_
C_5_S_2_S	4CaO·2SiO_2_·CaSO_4_
C_4_AF	4CaO·Al_2_O_3_·Fe_2_O_3_
C_4_A_2.85_Fe_1.5_S	3CaO·2.85Al_2_O_3_·1.5Fe_2_O_3_·CaSO_4_
AFtAFm	3CaO·Al_2_O_3_·3CaSO_4_·32H_2_O3CaO·Al_2_O_3_·CaSO_4_·12H_2_O
